# Anaphylactic reaction to gonadotropin‐releasing hormone analogues: a pediatric case report

**DOI:** 10.1002/ccr3.1550

**Published:** 2018-05-23

**Authors:** Franco Ricci, Maurizio de Martino, Stefano Stagi

**Affiliations:** ^1^ Department of Health Sciences Anna Meyer Children's University Hospital University of Florence Florence Italy

**Keywords:** Anaphylactic reactions, gonadotropin‐releasing hormone analogues, precocious puberty

## Abstract

Gonadotropin‐releasing hormone (GnRH) analogues represent the gold standard treatment for precocious puberty. Allergic‐type reactions in children due to this type of treatment are very rare. However, caregivers should be aware of the potential GnRH analogue systemic reactions.

## Introduction

Gonadotropin‐releasing hormone analogues are the treatment of choice for idiopathic central precocious puberty. A very few reports of anaphylactic reactions due to this treatment are reported in the current literature. In this report, we describe a pediatric patient with idiopathic central precocious puberty, admitted to our hospital for anaphylactic reactions occurred after i.m. administration of triptorelin.

Administration of gonadotropin‐releasing hormone (GnRH) analogues is the treatment of choice in children with idiopathic central precocious puberty 1. Anaphylactic reactions in patients receiving this treatment are very rare. In the current literature, only few cases have been reported 2, 3, 4. We describe a girl with idiopathic central precocious puberty who showed an anaphylactic reaction after administration of GnRH analogues.

## Case Presentation

The patient, a 7‐year and 6‐month‐old girl, was admitted to our hospital for premature thelarche. Her previous clinical history was negative. Her height was 131.5 cm (+1.06 SDS), and breast development was Tanner stage 3. LHRH test showed a LH peak equal to 17.7 mUI/mL and a FSH peak equal to 8.3 mUI/mL. 17*β*‐estradiol level was 35 pg/mL; adrenal and thyroid functions and cancer markers (*α*‐fetoprotein, *β*HCG, CEA) were in the normal range. The pelvic ultrasonography showed the presence of transitional uterus and ovaries with increased dimension and normal echostructure. Bone age assessed with Greulich and Pyle method was advanced (9 years). Nuclear magnetic resonance imaging of the pituitary and brain was normal. Diagnosis of idiopathic central precocious puberty was performed. She started a gonadotropin‐releasing hormone (GnRH) analogue therapy with triptorelin i.m. 3.75 mg every 28 days, and after 6 months, a new LHRH test showed suppressed gonadotropin peaks (a LH peak equal to 0.67 mUI/mL and a FSH peak equal to 0.60 mUI/mL) confirming the efficacy of treatment.

At the age of 8 years and 4 months, the patient was admitted to our Emergency Department for the appearance of abdominal pain, burning sensation at neck and dyspnea occurred two hours after the last injection of i.m. triptorelin. At the examination, pruriginous whistles of about 20 mm were present on the whole body. Diagnosis of anaphylactic reactions was performed, and a treatment with systemic epinephrine and intravenous hydrocortisone was started with prompt resolution of symptoms. Skin prick tests performed the day after showed a positive result at very low dilution of the drug (1:10.000). As a result, therapy with triptorelin was discontinued. At 6 months of follow‐up, no substantial progress of puberty or other anaphylactic reactions have been observed.

## Discussion

Precocious puberty is among the most frequent endocrine disorders, due to idiopathic or organic diseases. The treatment with GnRH analogues aims to improve the statural prognosis preserving the patient's height potential and prevent psychological complications due to premature menarche. Allergic‐type reactions to this type of treatment are very rare. Anaphylaxis is a presumptive diagnosis, and this is not confirmed by any in vitro or in vivo tests. Where available tryptase measurements it can be useful in supporting the diagnosis, however a normal tryptase level does not rule out anaphylaxis diagnosis 5. To our knowledge, this is the first anaphylactic reaction due to i.m. GnRH analogues reported in a pediatric patient. It may represent a case of sensitization during previous exposure. Only one pediatric case was previously reported, but the reaction appeared after a subcutaneous depot of GnRH analogue administration 6. We believe that the caregivers should be aware of the potential GnRH analogue systemic reactions in order to avoid any possible dangerous situations for the patient.

## Conflict of Interest

The authors exclude any conflict of interests in the work presented here.

## Authorship

FR: drafted the manuscript. MM: reviewed the manuscript. SS: assisted the patient and reviewed the manuscript. All authors read and approved the final manuscript.
